# Advanced glycation end products accelerate rat vascular calcification through RAGE/oxidative stress

**DOI:** 10.1186/1471-2261-13-13

**Published:** 2013-03-05

**Authors:** Qin Wei, Xiaomei Ren, Yibo Jiang, Hong Jin, Naifeng Liu, Jie Li

**Affiliations:** 1 Department & Institute of Cardiology, Zhongda Hospital, Southeast University, Nanjing, Jiangsu 210009, P. R. China; 2 Department of Geratology, Zhongda Hospital, Southeast University, Nanjing, Jiangsu 210009, P. R. China; 3 Department of Cardiology, Taixing Hospital affiliated with Yangzhou University, Jiangsu 225400, P. R. China

**Keywords:** Diabetes mellitus, Advanced glycation end products, Vascular smooth muscle cells, Calcification, Oxidative stress

## Abstract

**Background:**

Arterial media calcification (AMC) is highly prevalent and is a major cause of morbidity, mortality, stroke and amputation in patients with diabetes mellitus (DM). Previous research suggests that advanced glycation end products (AGEs) are responsible for vascular calcification in diabetic patients. The potential link between oxidative stress and AGEs-induced vascular calcification, however, has not been examined.

**Methods:**

Male Wistar rats received a high fat diet for 8 weeks followed by a single dose of streptozotocin to induce DM (DM). Calcification was induced with Vitamin D3 and nicotine (VDN). We started VDN treatment at 1 week after the initial streptozotocin injection (DM+VDN). Age-matched rats were used as controls (CON). Metabolic parameters, aortic calcium content, alkaline phosphatase (ALP) protein, malondialdehyde (MDA) content, Cu/Zn superoxide dismutase (SOD) activity, aorta receptor for advanced glycation end products (RAGE) and aorta AGEs levels were measured. In vitro, vascular smooth muscle cells (VSMCs) were cultured with AGEs in DMEM containing 10 mmol·L^-1^ ß -glycerophosphate (ß-GP). Calcium content and ALP activity were used to identify osteoblastic differentiation and mineralization. Western blots were used to examine protein expression of Cu/Zn SOD, NADPH oxidase Nox1 and RAGE. In addition, the intracellular reactive oxygen species (ROS) generation was evaluated using fluorescent techniques with dihydroethidine (DHE) method.

**Results:**

The DM+VDN group showed a significant increase in aortic calcium content, levels of aorta AGEs, MDA content, ALP protein levels and RAGE expression, although Cu/Zn SOD activity decreased significantly. In vitro, enhanced Nox1, RAGE expression as well as the production of intracellular superoxide anions, and reduced expression of Cu/Zn SOD induced by AGEs were attenuated by the anti-RAGE antibody or a ROS inhibitor. Furthermore, the AGEs-stimulated ROS increase was also significantly inhibited by a SOD mimetic. Increased ALP activity and calcium deposition were also inhibited markedly by the ROS inhibitor and the anti-RAGE antibody.

**Conclusions:**

These results suggest that AGEs enhance vascular calcification partly through a RAGE/oxidative stress pathway.

## Background

Vascular calcification is a form of heterotopic calcification, that results in a decrease in elasticity and compliance of the vessel walls, and was previously thought to be a passive physicochemical process. Recent studies have demonstrated that arterial calcification is highly prevalent in patients with diabetes mellitus (DM) compared to the general population and is associated with increased morbidity, mortality, stroke and amputation rates [1-4]. Over recent years, several studies have revealed that vascular calcification is an actively regulated process that is similar to osteogenesis.

Advanced glycation end products (AGEs) derived from reducing sugars reaction non-enzymatically with amino groups of protein play an important role in the pathogenesis of numerous diseases, including diabetic complications, atherosclerosis and aging. AGEs are formed at an accelerated rate under diabetic states and during renal failure [5]. Recently, considerable evidences have suggested that AGEs might contribute to vascular calcification. AGEs have the ability to accelerate calcification in microvascular pericytes, which could contribute to the development of vascular calcification [6]. A study by Taki K et al. showed a positive correlation between serum AGEs levels and the severity of coronary artery calcification in hemodialysis patients [7]. Alexi Baidoshvilia et al. found that in DM patients, N (epsilon)-(carboxymethyl)lysine (CML), a main antigenic structure of non-cross linking AGEs, accumulated on calcification sites in degenerated aortic valves and in internal thoracic arteries [8]. Indeed, previous data showed that AGEs enhanced calcification in vascular smooth muscle cells (VSMCs) through the receptor for AGEs (RAGE) pathway [9,10]. Therefore, these results suggest that AGEs are responsible for vascular calcification in diabetic patients.

Vascular reactive oxygen species (ROS) contribute to vascular functional and structural alterations. At the cellular levels, ROS can result in the reduction of proliferation, apoptosis, cell cycle arrest, and modulation of differentiation [11-13]. Previous study suggested that hydrogen peroxide or xanthine/xanthine oxidase increased intracellular oxidative stress and enhanced osteoblastic differentiation of vascular cells, as demonstrated by their analysis of alkaline phosphatase (ALP) activity and mineralization [14]. Indeed, the engagement of RAGE with AGEs can induce oxidative stress in VSMCs, thereby playing an important role in the development and progression of many diseases [15]. The potential link between oxidative stress and AGEs-induced vascular calcification, however, has not been examined.

Given these findings, we hypothesized that AGEs accelerated vascular calcification through the RAGE/oxidative stress pathway in the present study.

## Methods

### Model preparation

**Figure 1:**
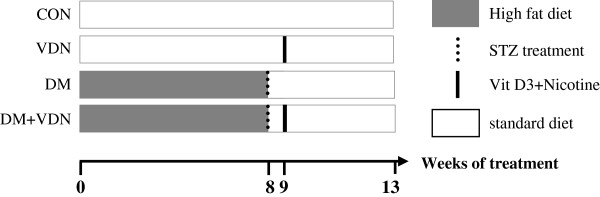
**Schematic representation of chronic treatments in rats.** Open bars represent a standard chow diet and gray bars represent a high fat diet. The vertical dashed lines represent the injection of streptozotocin. The vertical solid lines represent the vitamin D3 and nicotine treatment.

The DM accompanied with arterial media calcification (AMC) model was prepared as described previously [16]. All experiments were approved by the Animal Care and Research Committee of Southeast University, and all animal procedures were performed in accordance with the Guidelines of Animal Experiments from the Committee of Medical Ethics, the National Health Department of China (1998). Briefly, six-week old male Wistar rats (160–180 g, Experimental Animal Center in Shanghai, China) were divided into four groups. The first group was the DM group, and was fed a high fat diet (HFD, 50% carbohydrates, 30% fat (20% lard and 10% soybean oil), 11% protein, 2.5% cholesterol, 6.5% fiber and other ingredients) (Animal Center, Health Science Center, Southeast University) for 8 weeks, followed by a single dose of streptozotocin (STZ, 25 mg·kg^-1^, intraperitoneally, Sigma). Animals were considered to be diabetic when the blood glucose levels were over 16.7 mmol·L^-1^. All of the rats were maintained on a standard chow for 1 week after the start of the STZ administration. A portion of the diabetic rats were then switched to treatment with a vehicle buffer (0.1 mol·L^-1^ sodium citrate buffer, pH 4.5) and a standard chow diet (60% carbohydrates, 22% protein, 10% fat, 8% fiber and other ingredients) (n = 10). For the second group (the DM+VDN group), half of the diabetic rats were treated with a single dose of vitamin D3 (300 000 U/kg, intramuscularly, Sigma) and nicotine (25 mg·kg^-1^, 5 ml·kg^-1^, PO, Sigma) to induce AMC (n = 10). Nicotine administration was repeated at 7 p.m. on the same day. For the third group (the VDN group), 15-week old healthy rats were fed with a standard chow diet and were administered with vitamin D3 and nicotine (n = 10). Rats were then allowed to recover for 8 weeks. The CON group included age-matched rats that were only given a vehicle buffer and a standard rat chow diet (n = 10). Please see Figure 1 for a depiction of the treatment protocols.

### Metabolic parameters and specimen preparation

Rats were starved for twelve hours before sacrifice. All rats were anesthetized intraperitoneally with sodium pentobarbital (65 mg·kg^-1^). Blood samples were collected for measurement of plasma variables. Measurement methods for total cholesterol (TC), triglyceride (TG) concentrations, plasma glucose and insulin were described previously [16].

The thoracic aortas were isolated. A portion of the thoracic aortas were frozen at −80°C for subsequent calcium content and ALP protein expression.

### Assessment of malondialdehyde content and superoxide dismutase activity in aortas and serum samples

Prior to sacrificing the rats, serum samples were collected in vials for analysis of malondialdehyde (MDA) content and Cu/Zn superoxide dismutase (SOD) activity (Nanjing Jiancheng Biotechnology Institute, China). After sacrifice, a portion of the fresh aortas were collected in a buffer solution and were subsequently homogenized. Next, the homogenized tissue was centrifuged and supernatants were collected for the analysis of MDA content and Cu/Zn SOD activity. MDA content and Cu/Zn SOD activity were measured as described previously [17]. Briefly, MDA content was detected by the thiobarbituric acid method with a maximal absorbance at 532 nm, and Cu/Zn SOD activity was measured by the xanthine oxidase method with an absorbance at 550 nm by spectrometry.

### Determination of aorta AGEs

The levels of AGEs were analyzed by a fluorescent method. Supernatants from aorta homogenates were measured on a Hitachi 650–60 fluorescence spectrometer with excitation and emission wavelengths of 380 nm and 452 nm, respectively. Standard curves created with different concentrations of AGE-BSA (0, 1, 5, 10, 50, 100 μg·mL^-1^) were used to determine the linear range of the assays. For these assays, 1U of AGEs was equivalent to 1 μg·mL^-1^ of AGE-BSA.

### Preparation of AGE-BSA

Briefly, bovine serum albumin (BSA) and D-glucose were dissolved in sodium phosphate buffer (PBS). The final concentrations were 50 mmol·L^-1^ of glucose and 5 g·L^-1^ of BSA. The solution was sterilized by ultrafiltration, then was incubated at 37°C for 90 days, and finally was dialyzed against PBS. As a control, BSA was incubated in parallel without D-glucose. No endotoxin was detectable in these preparations.

### Induction of calcification of rat VSMCs in vitro

VSMCs were isolated from one-week old Sprague–Dawley rat thoracic aortas (Experimental Animal Centre, Southeast University, Nanjing, China) and cultured as described previously [9]. Passages of 3–5 VSMCs were used for the study. At sub-confluence, calcification of the cells was induced in DMEM with 1% FCS in the presence of 10 mM β-glycerophosphate (β-GP, Sigma, USA). This medium was replaced twice a week. Cells were harvested for analysis at Day 3 of the treatment. In separate experiments, sub-confluent VSMCs cultured in six-well culture plates were pre-incubated with a neutralizing antibody to RAGE (100 μg·mL^-1^, R&D) or 10 μmol·L^-1^diphenyleneiodonium (DPI, Sigma), an inhibitor of ROS for 2 h. Next, AGE-BSA was added to the medium containing 10 mmol·L^-1^ of β-GP for 24 h prior to the measurement of gene and protein expression, or for 72 h prior to the measurement of calcium content. For all data shown, individual experiments were repeated at least three times.

### Quantification of calcium deposition

Analysis of calcium content in rat thoracic aortas was performed according to previously described procedures [18]. Briefly, portions of aortas were dried at 55°C and calcium was extracted overnight at 4°C using 10% formic acid. Cells were decalcified with 0.6 M HCl for 24 h. The calcium content in acid extracts was examined colorimetrically by the O-Cresolphthalein Complexone method (Calcium kit; Sigma) [10,19]. After decalcification, cells were washed three times with PBS and solubilized with 0.1 M NaOH containing 0.1% SDS. A BCA assay kit (Pierce) was used to determined protein concentration. Aorta calcium content was expressed as μg/mg of dry tissue, and cell calcium content was normalized to total protein content.

### Examination of ALP activity

Cellular proteins were solubilized with 1% Triton X-100 in 0.9% NaCl. After centrifugation, the supernatants were assayed for ALP activity by using an ALP Assay Kit (Sigma, USA) as described previously [20]. One unit was defined as the activity producing 1 nmol of P-nitrophenol for 30 min. The results were normalized to total cellular protein levels as previously described [21].

### Western blot analysis

Total proteins of aortas and cells were extracted in the RIPA lysis buffer (Beyotime, China). After centrifugation, supernatants were collected for the determination of protein concentrations. Equal protein samples were loaded on a 10% SDS–PAGE gel and transferred to PVDF membranes (Bio-Rad). The membranes were blocked with 4% milk-TBST, incubated overnight with the primary antibody, washed with TBST, and then incubated with the secondary horseradish peroxidase-labeled antibody (Santa Cruz, USA, dilution 1:1000). Immunodetection was performed with the Enhanced Chemiluminescence Kit (Amersham Biosciences, Piscataway, NJ). The following antibodies were used: anti-RAGE monoclonal antibody (diluted 1:250, R&D), anti-ALP monoclonal antibody (diluted 1:500, Santa Cruz Biotechnology Inc), anti-Cu/Zn SOD monoclonal antibody (diluted 1:500, Santa Cruz Biotechnology Inc), and anti-Nox1monoclonal antibody (diluted 1:500, Santa Cruz Biotechnology Inc).

### Measurement of intracellular ROS generation

VSMCs calcification was induced with different concentrations of AGE-BSA for 2 h to analyze ROS. In separate experiments, VSMCs cultured in 24-well culture plates were pre-incubated with a neutralizing antibody to RAGE, DPI, or 100 μmol·L^-1^ tempol ( a SOD mimetic, R&D) for 2 h, followed by treatment with 100 mg·L^-1^AGE-BSA for another 2 h to determine ROS levels. ROS generation was evaluated using the fluorescent technique with dihydroethidine (DHE, Genmed Scientifics Inc. USA) according to the manufacturer’s instructions. VSMCs were incubated with DHE (diluted 1:100 with dilution reagents) for 20 min at 37°C in the dark and were then washed with PBS. Fluorescence was determined using a microscope (Nikon, Japan) at excitation and emission wavelengths of 540 nm and 590 nm, respectively. Fluorescence intensity was semi-quantitatively analyzed by NIS-Elements F 3.0.

### Immunohistochemistry

Immunohistochemistry was performed to detect RAGE. Briefly, after dewaxing and hydration of sections, tissue slides were treated with 3% H_2_O_2_ for 10 min, then were incubated with the primary antibody against RAGE (R&D, 1:20) overnight at 4°C, followed by incubation with the secondary antibody conjugated to horseradish peroxidase. The antibodies were detected by the diaminobenzidine method which produces a brown color. Adjacent sections treated with non-immune IgG provided controls for antibody specificity. The area of the aorta with brown staining was quantified. The analysis was performed blindly by two examiners for the same slides.

### Von kossa staining

The presence of mineral deposits was confirmed by Von kossa staining. Paraffin sections from aortas were dewaxed and hydrated. The slices were immersed in 1% silver nitrate for 30 min under an intense sunbeam, and were then washed 3 times with deionized water. Subsequently, 5% sodium thiosulfate (Sigma) was added for 5 min to remove un-reacted silver. The calcium phosphate salts were visualized as a black staining.

### Statistical analysis

Data were presented as the mean ± SD. A one-way ANOVA followed by a post hoctest and chi-square test were performed for statistical comparisons. P< 0.05 was considered to be significant.

## Results

### Metabolic parameters

After completion of 8 weeks of high fat diet feeding, serum triglycerides and cholesterol concentration measurements showed a significant difference between HFD rats and CON rats (TG: 2.25±0.25 mmol·L^-1^vs 0.48±0.05 mmol·L^-1^, TC: 6.22±0.57 mmol·L^-1^vs 2.74±0.32 mmol·L^-1^, respectively, p<0.05). At the end of the experiment, as shown in Table 1, these diabetic rats showed weight loss, low insulinemia and high glycemia. The addition of VDN accelerated this tendency in diabetic rats. Weight loss was not observed in the VDN group. In addition, no significant differences were detected regarding serum triglycerides and cholesterol concentrations among all groups, suggesting that VDN treatment alone did not affect serum lipids and that normal lipid metabolism in the DM treatment groups maybe associate with weight loss.

**Table 1:** Metabolic parameters in the experimental groups

**Groups**	**n**	**Weight (g)**	**Glu (mmol·L**^**-1**^**)**	**Ins (ng·mL**^**-1**^**)**	**Cho (mmol·L**^**-1**^**)**	**TG (mmol·L**^**-1**^**)**	**HbA1c(%)**
CON	10	530 ± 20	5.53 ± 0.26	0.75 ± 0.12	2.21 ± 0.19	0.47 ± 0.05	7.1 ± 1.1
DM	10	470 ± 15^*#^	17.21 ± 0.95^*#^	0.58 ± 0.11^*#^	2.09 ± 0.22	0.51 ± 0.11	16.5 ± 2.7^*#^
VDN	10	521 ± 16	5.26 ± 0.46	0.69 ± 0.13	2.12 ± 0.21	0.42 ± 0.03	8.2 ± 1.3
DM+VDN	10	422 ± 17^*#^	25.61 ± 1.92^*#^	0.32 ± 0.05^*#^	1.99 ± 0.18	0.49 ± 0.04	23.4 ± 3.5^*#^

*p< 0.05 vs without VDN. #p< 0.05 vs VDN group. Cho, cholesterol. Glu, glucose. Ins, insulin. TG, triglyceride.

### Medial calcification in thoracic aortas

**Figure 2:**
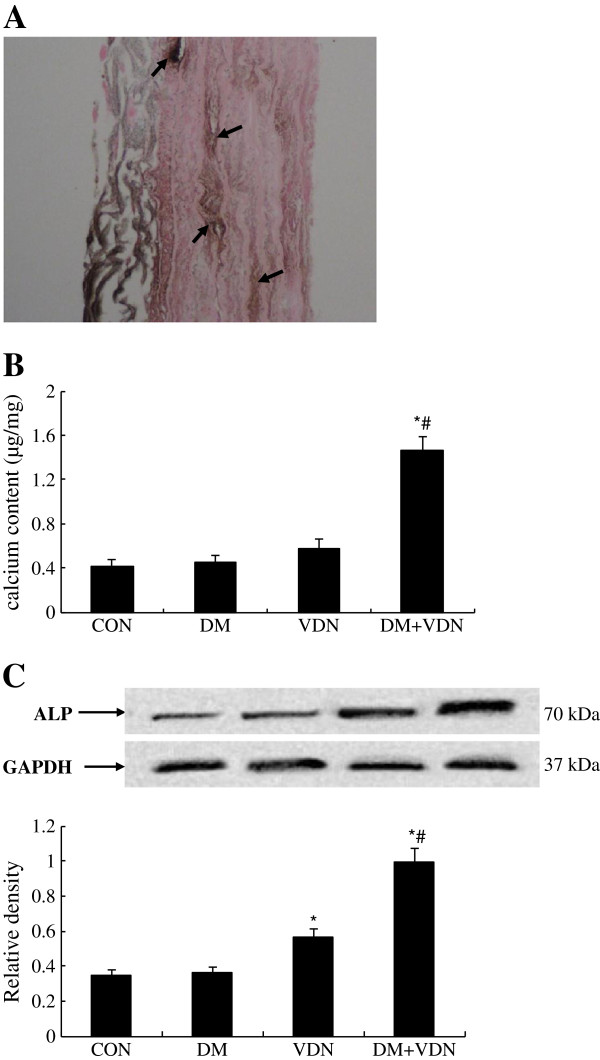
**Diabetes accelerated rat aorta calcification. (A)** Von kossa staining (black calcium particles, ×200). **(B)** Aorta calcium content. **(C)** ALP protein expression. Values represent the mean ± SD (n=10). * p<0.05 versus control group, # p<0.05 versus VDN group.

Three weeks of VDN treatment did not produce calcification in the aorta (data not shown). Minimal calcification was expected in the aorta media when rats were treated with VDN for four weeks. The Von kossa staining revealed extensive calcium deposits in the arterial media of the DM+VDN group (Figure 2A).To study the acceleration of vascular calcification, rats were sacrificed four weeks after VDN treatment. As shown in Figure 2B, only the DM and VDN treatments did not cause a significant elevation of calcium content in the aortas. Calcium content in the aortas of DM+VDN rats, however, were significantly increased (p<0.05). Aorta ALP protein expression was significantly increased in both the VDN group and DM+VDN group compared to the DM and control groups. Moreover, aorta ALP protein expression in the DM+VDN group was significantly higher than those found in the VDN group (p<0.05, Figure 2C).

### SOD activity and MDA content in serum samples and thoracic aortas

**Figure 3:**
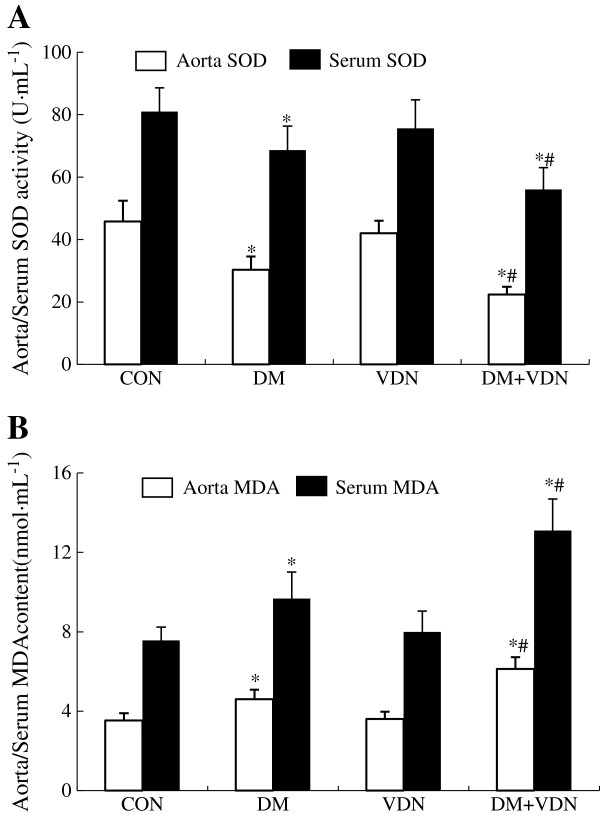
**MDA content and Cu/Zn SOD activity in aorta and serum. (A)** Rat aorta and serum SOD activity. **(B)** Rat aorta and serum MDA content. Values represent the mean ± SD (n=10). * p<0.05 versus control group, # p<0.05 versus VDN and DM group.

Compared to the CON group, Cu/Zn SOD activity and MDA content in aortas and serum samples of VDN group (p>0.05, Figure 3) showed no significant differences. In the VDN+DM and DM groups, when compared to the CON and VDN groups (p<0.05, Figure 3), the production of MDA in aortas and serum samples was significantly increased, and Cu/Zn SOD activity was decreased compared to the DM and VDN groups (p<0.05, Figure 3). Moreover, changes of SOD activity and MDA content were more significant in the DM+VDN group than in the DM group (p<0.05, Figure 3).

### Aorta AGEs levels and RAGE expression

**Figure 4:**
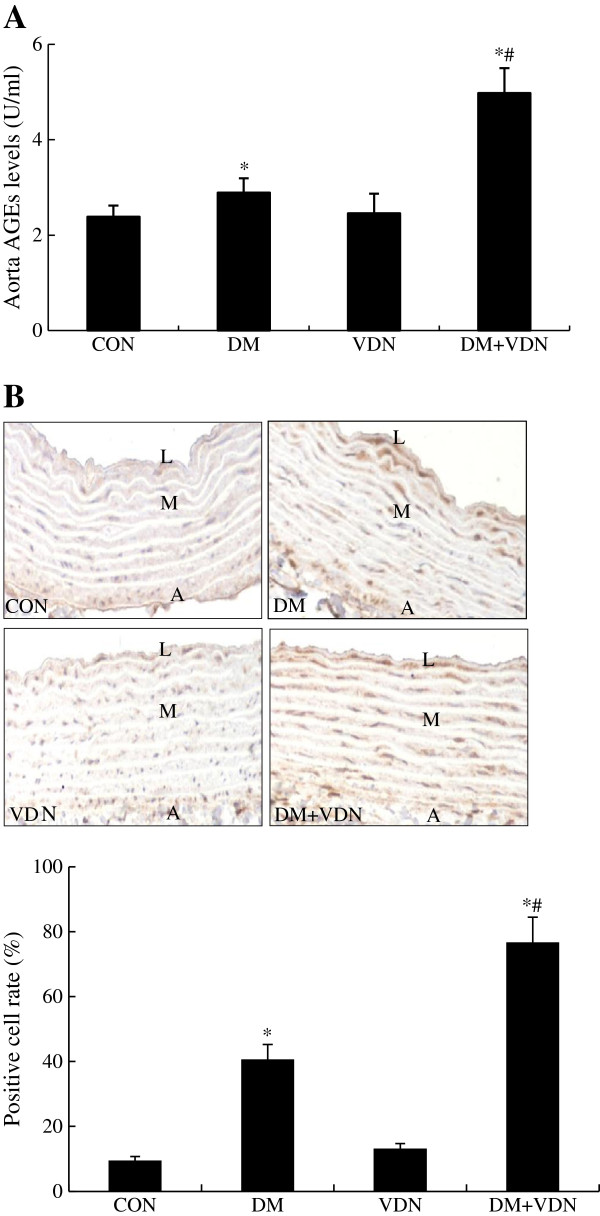
**The levels of aorta AGEs and aorta RAGE protein expression increased in DM+VDN rats. (A)** Aorta AGEs levels. **(B)** Aorta RAGE expression (×200). Values represent the mean ± SD. L: Lumen, M: Media, A: Adventitia.* p<0.05 versus control group, # p<0.05 versus VDN and DM group.

Immunohistochemical staining for RAGE in aortas revealed that RAGE appeared in the media. As shown in Figure 4, compared to the control group, RAGE expression increased significantly in the DM and DM+VDN groups (p<0.05). Moreover, RAGE expression was more significant in the DM+VDN group than in the DM group (p<0.05). However, an increase of RAGE expression in the VDN group was not detected. Aorta AGEs levels followed the pattern of RAGE expression in the different groups.

### Effect of AGEs on redox signaling

**Figure 5:**
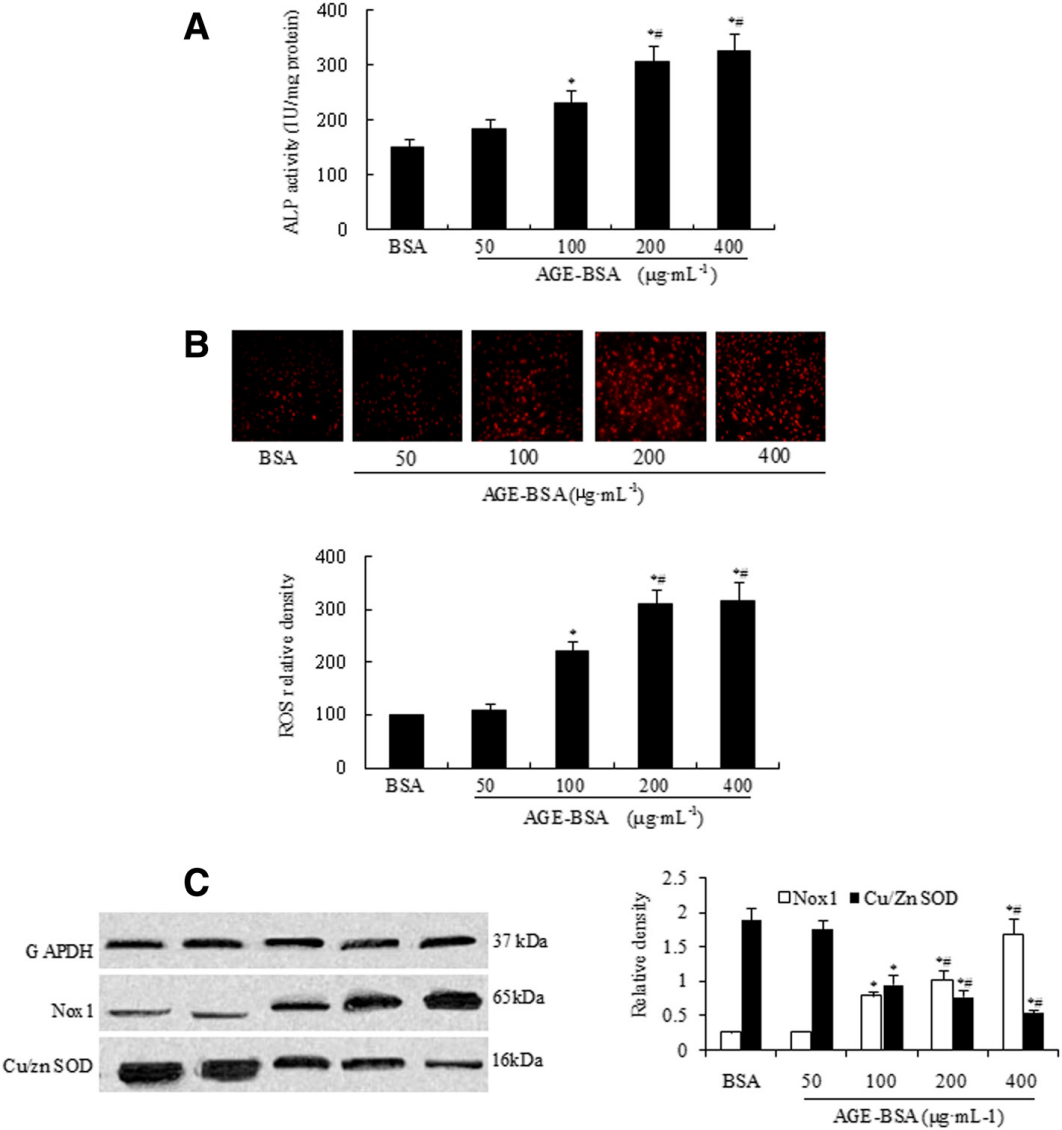
**Effects of AGEs on ALP activity, superoxide production, and protein expression of Nox1 and Cu/Zn SOD on calcification of rat VSMCs induced by β-GP.** VSMCs were treated with various concentrations of AGEs (BSA, 50, 100, 200, 400 μg·mL^-1^) for 24 h. **(A)** ALP activity. **(B)** Cellular levels of superoxide. **(C)** Protein expression of Nox1 and Cu/Zn SOD. These results were obtained from at least three independent measurements. * p<0.05 versus BSA, # p<0.05 versus 100 μg·mL^-1^AGE-BSA.

To understand the mechanism by which AGEs accelerate vascular calcification, in vivo, we further examined whether an imbalance of oxygen free radical production and destruction was involved in this process. As shown in Figure 5C and Figure 5A, AGEs significantly increased the levels of Nox1 protein and ALP activity in a dose-dependent manner in cultured VSMCs after 72 h of calcification induction (p<0.05). In addition, ROS production was also significantly increased compared to the control group (Figure 5B, p<0.05), whereas, Cu/Zn SOD protein expression was markedly suppressed (Figure 5C, p<0.05).

### Effect of RAGE blockade and ROS inhibition on calcification and redox signaling in VSMCs

**Figure 6:**
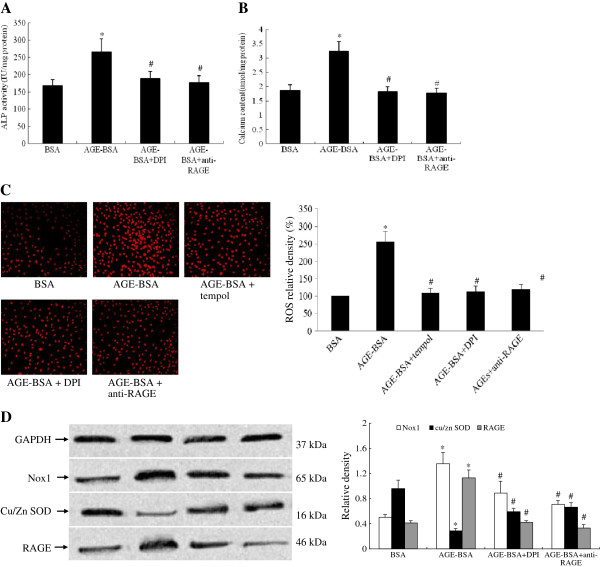
**Effect of RAGE blockade and ROS inhibition on osteoblastic differentiation and oxidative stress signaling pathways in rat VSMCs calcification accelerated by AGEs. (A)** ALP activity. **(B)** Calcium content. **(C)** ROS generation (×200). **(D)** Nox1, RAGE and Cu/Zn SOD protein expression. Data were from three separate experiments. * p<0.05 vs BSA, # p<0.05 vs AGE-BSA.

Pretreatment of VSMCs with the neutralizing antibody to RAGE or DPI for 2 h reduced the increase of ALP activity induced by AGEs after a 24 h cultivation period (Figure 6A, p<0.05). Figure 6B shows the effect of the RAGE blockade or DPI inhibition on calcium content, indicating that the anti-RAGE antibody and DPI significantly decreased calcium content following induction by 100 mg·L^-1^ of AGEs after a 72 h incubation. The increased ROS generation induced by AGEs was significantly eliminated by 2 h-treatment with DPI, anti-RAGE or tempol (Figure 6C, p<0.05). RAGE and Nox1 protein expression induced by AGEs were significantly inhibited, however, Cu/Zn SOD protein expression increased significantly (Figure 6D, p<0.05).

## Discussion

Although vascular calcification is a strong predictor of diabetes-related cardiovascular events [4], the underlying mechanism of this observation remains unclear. Elucidation of these mechanisms is important for the establishment of new therapies to prevent vascular calcification. Increasing evidence has suggested that vascular calcification is an active and regulated process. Many cardiovascular factors, including inorganic phosphate and oxidized LDL, accelerate vascular calcification [22-24]. AGEs are among the factors responsible for diabetic vascular complications [25,26], and RAGE is the most well characterized receptor for AGEs [27]. AGEs are formed at an accelerated rate under diabetic states. In the present study, we first selected a rat model of diabetes that shared similarities with type 2 diabetes in human population. In our model, the administration of the HFD induced an insulin resistance state (data was not shown), which is consistent with the results obtained by other groups working with rats [18,28]. The administration of a low dose of STZ (25 mg/kg) aimed at slightly reducing the β cell function to obtain hyperglycemia, since a 30 mg/kg dose of STZ administration insulin deficiency results in insulin deficiency and diabetes could be thought as type 1 [29].

In our previous study, we demonstrated that AGEs accelerated vascular calcification by promoting osteoblast-like differentiation of rat VSMCs via the RAGE pathway [9]. Tanikawa T et al. reported similar findings [10]. The present study demonstrates that the degree of arterial calcification, the levels of aorta AGEs and RAGE expression are significantly increased in DM+VDN rats (Figure 2 and Figure 4). In addition, hyperglycemia was more prominent in DM+VDN rats (Table 1). Although high glucose levels promoted calcification of VSMCs [30], other studies further supported the pathological role of AGEs in vascular calcification. Tanikawa T et al. found that AGEs induced calcification of vascular smooth muscle cells irrespective of glucose concentration. Furthermore, calcification of VSMCs induced by diabetic serum was inhibited by RAGE blockade [10]. The relationship between cardiovascular mortality of patients with diabetes and vascular calcification was observed regardless of glycemic control and known duration of diabetes [31]. Additionally, our data did not show an increase in serum lipid levels among all groups [16]. This apparently normal lipid metabolism should not influence our results, as medial calcification is not associated with lipid deposition. These results suggest that AGEs might accelerate vascular calcification that is initiated by VDN and that AGEs are among the factors responsible for vascular calcification in diabetes. However, the mechanism remains unknown in these previous studies.

Oxidative stress results from an increase in the production of ROS, notably superoxide anions. The ROS act as second messengers and play an important role in the development of diabetic vascular complications. AGEs exaggerate the vascular damage that is subjected to increased oxidative stress [32,33]. In addition, several researches have suggested that oxidative stress plays a role in vascular calcification. F. T. Tang et al. reported that hypercholesterolemia accelerated vascular calcification induced by excessive vitamin D via oxidative stress [34]. H_2_O_2_ promotes a phenotypic switch of VSMC from a contractile to an osteogenic phenotype [35]. We therefore hypothesized that oxidative stress may be the downstream signal of AGE/RAGE accelerating calcification.

To investigate the role of oxidative stress in the rat vascular calcification model, we measured MDA, which serves as a lipid peroxidation marker and has been validated in several mouse models regarding oxidative stress. We also examined SOD, which acts as the first line of defense against oxygen free radical-mediated damage by catalyzing the dismutation of superoxide anions. In the present study, we found that serum and aorta MDA content in DM+VDN rats increased significantly, however, Cu/Zn SOD activity decreased markedly (Figure 3). Together with increased aorta AGEs levels and RAGE expression in DM+VDN rats (Figure 4), our findings suggest that at least one mechanism by which AGEs augment vascular calcification involves promoting oxidative stress, although many factors are clearly involved in the initiation and progression of vascular calcification.

Next, we examined markers of oxidative stress in cultured VSMCs. Consistent with previous studies in various cell lines, our results also showed AGEs linked to enhanced oxidative stress in calcified VSMCs. We found a marked increase in intracellular O_2_^-^ production in calcified VSMCs induced by AGEs (Figure 5B). Inhibition of ROS with DPI and RAGE blockade reduced O_2_^-^ production considerably (Figure 6C), and attenuated the calcification that we observed in cultured VSMCs (Figure 6A 6B). These results showed that ROS is involved in AGEs-induced calcification. NADPH oxidase is the major source of ROS production. There are five known forms of NADPH oxidase, which are classified by the presence of different Nox isoforms (Nox1-5) [36]. Previously, it has been shown that agonist-induced ROS production in VSMCs is mediated by Nox1 [37] and AGEs accelerates dysfunction in VSMCs, particularly under conditions of increased Nox1 expression [38,39]. The above-mentioned observations and studies suggested that Nox1 could be a mediator of ROS production in VSMCs calcification induced by AGEs. We therefore sought to investigate Nox1 expression in calcified VSMCs induced by AGEs. Our results showed that Nox1 expression increased (Figure 5C). And ROS inhibition with DPI and RAGE blockade markedly reduced Nox1 expression (Figure 6D). These findings support that AGEs-induced calcification in VSMCs is mediated by oxidative stress, at least in part, involving Nox1. Of course, other Nox isoforms maybe also play a role in AGEs-induced ROS production in calcified VSMCs. Further research about the role of all Noxs in vascular calcification induced by AGEs is necessary in the future.

Conversely, SOD is a primary cellular defense against ROS, and the predominant activity of SOD in peripheral vessels is attributed to Cu/Zn SOD [40]. We observed a significant reduction in Cu/Zn SOD expression (Figure 5C) accompanied with a marked increase in cellular O2- production in calcified VSMCs induced by AGEs (Figure 5B). And ROS inhibition with DPI and RAGE blockade increased markedly Cu/Zn SOD expression (Figure 6D).These results show that vascular calcification induced by AGEs is mediated by oxidative stress. Further supporting evidence comes from our findings that SOD mimetic inhibited ROS production induced by AGEs (Figure 6C).

### Limitations of the study

In vivo, our data indicated that AGE/RAGE/oxidative stress axis was associated with diabetic vascular calcification. We further confirmed this effect of AGEs on calcification and the mechanisms involved in vascular calcification in vitro. There is no direct in vivo evidence that AGEs accelerate vascular calcification or that oxidative stress mediates this effect. We did not investigate other Nox and SOD isoforms in addition to Nox1 and Cu/Zn SOD. Nox2 and Nox4 have recently been implicated in superoxides. Mitochondrial derived ROS may be involved in vascular calcification accelerated by AGEs.

## Conclusion

In summary, this study demonstrates that AGEs might enhance vascular calcification induced by Vitamin D3 and nicotine in rats via an oxidative stress pathway. This hypothesis is further supported by in vitro results showing that blockage of RAGE and ROS inhibition attenuates the acceleration of rat VSMCs calcification by AGEs. These results indicate that inhibition of the AGE/RAGE/oxidative stress pathway could potentially be an effective therapy for the prevention of vascular calcification in diabetic patients.

## Abbreviations

AGEs: Advanced glycation end products; RAGE: Receptor for advanced glycation end products; MDA: Malondialdehyde; ROS: Reactive oxygen species; VSMCs: Vascular smooth muscle cells; SOD: Superoxide dismutase; ALP: Alkaline phosphatase; NADPH: Nnicotinamide-adenine dinucleotide phosphate; DPI: Diphenylene iodonium; β-GP: β-glycerophosphate; DHE: Dihydroethidine; HFD: High fat diet; TC: Total cholesterol; TG: Triglyceride; MAC: Arterial media calcification; DM: Diabetes mellitus; BSA: Bovine serum albumin; XO: Xanthine oxidase.

## Competing interests

The authors have declared that no competing financial or any other kind of personal interests exist in this paper.

## Authors’ contributions

QW was the principal investigator, involved in designing the study, biochemistry detection, performing the statistical calculations and writing the manuscript. XMR was involved in the animal model preparation. YBJ, HJ and JL contributed to cell culture and parts of the biochemistry detection. NFL provided expertise in the research design and plan coordination. All authors participated in writing the final version of the manuscript. All authors read and approved the final manuscript.

## Pre-publication history

The pre-publication history for this paper can be accessed here:

http://www.biomedcentral.com/1471-2261/13/13/prepub
